# Nonequilibrium Enhances Adaptation Efficiency of Stochastic Biochemical Systems

**DOI:** 10.1371/journal.pone.0155838

**Published:** 2016-05-19

**Authors:** Chen Jia, Minping Qian

**Affiliations:** 1 Beijing Computational Science Research Center, Beijing, China; 2 Department of Mathematical Sciences, The University of Texas at Dallas, Richardson, Texas, United States of America; 3 School of Mathematical Sciences, Peking University, Beijing, China; Tata Institute of Fundamental Research, INDIA

## Abstract

Adaptation is a crucial biological function possessed by many sensory systems. Early work has shown that some influential equilibrium models can achieve accurate adaptation. However, recent studies indicate that there are close relationships between adaptation and nonequilibrium. In this paper, we provide an explanation of these two seemingly contradictory results based on Markov models with relatively simple networks. We show that as the nonequilibrium driving becomes stronger, the system under consideration will undergo a phase transition along a fixed direction: from non-adaptation to simple adaptation then to oscillatory adaptation, while the transition in the opposite direction is forbidden. This indicates that although adaptation may be observed in equilibrium systems, it tends to occur in systems far away from equilibrium. In addition, we find that nonequilibrium will improve the performance of adaptation by enhancing the adaptation efficiency. All these results provide a deeper insight into the connection between adaptation and nonequilibrium. Finally, we use a more complicated network model of bacterial chemotaxis to validate the main results of this paper.

## Introduction

**Fig 1:**
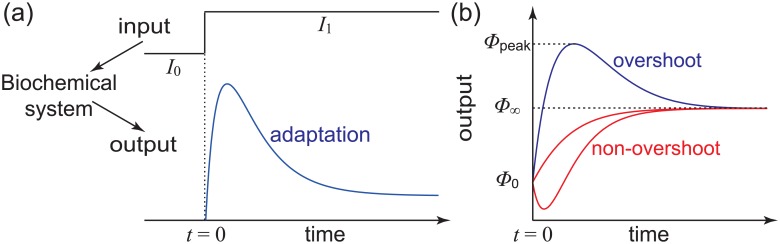
Adaptation and overshoot in biochemical systems. (a) Adaptation. (b) Overshoot and non-overshoot.

Adaptation is a crucial biological function by which a living system becomes better suited to its environment [1–5]. Specifically, adaptation refers to the ability of a biochemical system to recover to its initial state after a transient response to an abrupt environmental change. An intuitive description of adaptation is illustrated in Fig 1(a). In response to a step increase of the input stimulus, the output of the system undergoes a transient increase to a peak followed by a decrease to a lower plateau [3, 4].

Another important concept associated with adaptation is overshoot. Overshoot refers to the dynamic phenomenon that the output of a system varies with time in a non-monotonic way and exceeds both its initial and steady-state values over a certain period of time (see Fig 1(b)). In general, an adaptation system will perform overshoot. Thus the concepts of adaptation and overshoot are closely related and interconnected.

Adaptation is observed in a wide variety of sensory systems and its examples include the chemotaxis of bacteria [6–8], the osmotic shock response in yeast [9], the odor response in mammalian olfactory receptors [10, 11], the light response in mammalian retinal rods [12], the mechanical stress response in mammalian mechanoreceptors [13, 14], the calcium response in mammalian inositol trisphosphate receptors [15, 16] and ryanodine receptors [17, 18], and the hormone response in mammalian hormone receptors [19, 20]. The benefits of adaptation are well documented. It allows a biochemical system to detect the environmental fluctuations more rapidly and more sensitively, and thus protects the system from irreversible damages caused by unfavorable conditions.

To understand how adaptation is achieved, a large number of Markov models for various sensory systems have been proposed over the years [17, 21–27]. In the early work, one of the most influential models is the Markov model based on ligand-induced receptor modification proposed by Segel, Goldbeter, and coworkers, which has been successfully applied to describe adaptation of many chemoreceptors such as ryanodine receptors and hormone receptors [21–24]. Recently, significant progresses have been made on the biochemical feedback networks of adaptation systems [1–3]. Among these studies, Ma et al [3] searched all three-node feedback networks and found that adaptation is most likely to be achieved in two types of networks: the negative feedback loop or the incoherent feedforward loop.

In recent years, another exciting topic on adaptation is the relationship between adaptation and nonequilibrium in stochastic biochemical systems [5, 28–31]. Recall that if the surrounding conditions remain constant, a biochemical system will reach a steady state for which two classes must be distinguished. In an equilibrium state, the detailed balance condition is satisfied. In a nonequilibrium steady state (NESS), however, the breaking of detailed balance indicates that the system is externally driven with concomitant energy dissipation [32]. Inspired by [3], Lan et al [5] studied sensory systems with a negative feedback network. They found that the negative feedback mechanism must break detailed balance and they also obtained a relation that bridges the energy dissipation rate, adaptation speed, and adaptation accuracy. In addition, Jia et al [30] found that in some particular Markov models, nonequilibrium is even a necessary condition for adaptation.

However, we are now faced with two seemingly contradictory statements. In the early work, equilibrium Markov models were widely used to describe adaptation in various sensory systems [17, 21–27]. It has been shown that an equilibrium model based on ligand-induce receptor modification can achieve accurate adaptation [21–24]. These results appear to contradict recent studies that emphasize the necessity of nonequilibrium and energy dissipation for adaptation. This inconsistency is the motivation of this paper.

In the present paper, we provide an explanation of this inconsistency based on Markov models with relatively simple networks. Previous studies have shown that a biochemical system may perform three different types of dynamic behavior: non-adaptation, simple adaptation, and oscillatory adaptation [33]. In simple Markov models, we show that as the nonequilibrium driving becomes stronger, the system will undergo a phase transition along a fixed direction: from non-adaptation to simple adaptation then to oscillatory adaptation, while the transition in the opposite direction is forbidden. During the phase transition, the system may skip a particular type of dynamic behavior. If the non-adaptation phase is skipped, then the system will transition directly from simple adaptation to oscillatory adaptation. This suggests that although adaptation could be observed in equilibrium systems, it tends to occur in systems with a strong nonequilibrium driving.

In addition, we also study the effects of nonequilibrium on the performance of adaptation, which is usually characterized by two quantities: the adaptation sensitivity and accuracy [3, 5]. In this paper, we define another indicator named as adaptation efficiency. We find that as the nonequilibrium driving becomes stronger, both the three indicators will increase. This shows that nonequilibrium will improve the performance of adaptation. Incidentally, we also find that in realistic biochemical systems, it is difficult to distinguish oscillatory adaptation from simple adaptation by the experimentally observed output curve. This explains why adaptation with oscillatory dynamics is rarely reported in the documented cases.

To validate the main results of this paper, we study the relationship between adaptation and nonequilibrium based on the sensory network model of the *E. coli* chemotactic sensory system [5, 31, 34] by both theoretical and numerical methods. The numerical results coincide perfectly with the main conclusions of this paper.

## Model and Concepts

### Markov model

Markov jump processes are widely used to model various stochastic biochemical systems such as chemical reaction networks [35], single-molecule enzyme kinetics [36], allostery of proteins or ion channels [33], and phenotypic switching of cell populations [37]. In this paper, we consider a biochemical system modeled by a Markov jump process with generator matrix *Q* = (*q*_*ij*_), where *q*_*ij*_ with *i* ≠ *j* denotes the transition rate from state *i* to *j* and *q*_*ii*_ = −∑_*j* ≠ *i*_ *q*_*ij*_. We assume that the system can exist in *N* different states, denoted by 1, ⋯, *N*. The implication of these states varies from systems to systems. Each state can represent a binding state of an enzyme molecule [36], a conformational state of a receptor molecule [33], or a phenotypic state of a cell population [37].

Let *p*(*t*) = (*p*_1_(*t*), ⋯, *p*_*N*_(*t*)) denote the probability distribution of the system, where *p*_*i*_(*t*) denotes the probability of the system being in state *i* at time *t*. Then the dynamics of the system is governed by the master equation

$$\begin{array}{c} \frac{dp(t)}{dt}=p\left(t\right)Q. \end{array}$$ (1)

In this paper, we assume that the matrix *Q* has *N* different eigenvalues, since any matrix can be approximated by such a matrix with arbitrarily high accuracy.

In applications, the system is always associated with an observable *g* = (*g*_1_, ⋯, *g*_*N*_). For instance, if the system models allostery of an ion channel, then the observable *g* is often chosen as *g*_*i*_ = 1 if *i* is an open state of the channel and *g*_*i*_ = 0 if *i* is a closed state of the channel. With these notations, the output *ϕ*(*t*) of the system is then the ensemble average of the observable *g*:

$$\begin{array}{c} \phi \left(t\right)=\sum_{i=1}^{N}p_{i}\left(t\right)g_{i}=p\left(t\right)g^{T}, \end{array}$$

where *g*^*T*^ denotes the transpose of *g*. By solving Eq (1), it is easy to see that the output *ϕ*(*t*) is nothing but a linear combination of exponential functions:

$$\begin{array}{c} \phi \left(t\right)=\sum_{i=1}^{N}c_{i}e^{\lambda_{i}t}, \end{array}$$

where *λ*_1_, ⋯, *λ*_*N*_ are all the eigenvalues of the matrix *Q* and *c*_1_, ⋯, *c*_*N*_ are *N* constants (see S1 Appendix:1). The famous Perron-Frobenius theorem in the matrix theory claims that one eigenvalue of the generator matrix *Q* must be zero and the real parts of other eigenvalues are all negative [38]. In the sequel, we always fix *λ*_*N*_ = 0 and thus the output *ϕ*(*t*) can be rewritten as

$$\begin{array}{c} \phi \left(t\right)=\phi_{\infty }+\sum_{i=1}^{N-1}c_{i}e^{\lambda_{i}t}, \end{array}$$ (2)

where *ϕ*_∞_ is the steady-state output of the system. It is easy to see that if *λ*_*i*_ is a real number, then *c*_*i*_ is also a real number, whereas if *λ*_*i*_ and *λ*_*j*_ are conjugate complex numbers, then *c*_*i*_ and *c*_*j*_ are also conjugate complex numbers (see S1 Appendix:1).

### Overshoot

Let *π* = (*π*_1_, ⋯, *π*_*N*_) and *μ* = (*μ*_1_, ⋯, *μ*_*N*_) denote the initial and steady-state distributions of the system, respectively. Then the initial output of the system is *ϕ*_0_ = ∑_*i*_ *π*_*i*_ *g*_*i*_ and the steady-state output of the system is *ϕ*_∞_ = ∑_*i*_ *μ*_*i*_ *g*_*i*_. Following the standard definition, we say that the system performs overshoot if the output first rises to a peak and then declines to a lower plateau (see Fig 1(b)). Mathematically, this is equivalent to saying that there exists a time *t* such that *ϕ*(*t*) is larger than both *ϕ*_0_ and *ϕ*_∞_.

In realistic biochemical systems, the output usually increases at small times and the steady-state output is usually larger than the initial output (see Fig 1(b)). Mathematically, these two conditions mean that *ϕ*′(0) > 0 and *ϕ*_∞_ ≥ *ϕ*_0_, where *ϕ*′(*t*) denotes the derivative of *ϕ*(*t*). In the following discussions, we always assume that these two conditions are satisfied.

### Adaptation

In practice, a sensory system usually has an input *I*, which represents a signal from the environment. In this case, both the generator matrix *Q* and the output *ϕ*(*t*) will depend on *I*. In response to a step increase of the input *I*, the output *ϕ*(*t*) of a sensory system usually undergoes a transient increase to a peak followed by a decrease to a lower plateau (see Fig 1(a)). The output recovery after a transient response to the input stimulus is called adaptation [3, 4, 31].

Let *I*_0_ and *I*_1_ denote the initial and final input levels, respectively, and we assume that the input level is elevated from *I*_0_ to *I*_1_ at time *t* = 0 (see Fig 1(a)). In response to the step increase of the input stimulus, the system is driven from a steady state to another one. The initial distribution *π* of the system is exactly the steady-state distribution for the input *I*_0_ and the final distribution *μ* is exactly the steady-state distribution for the input *I*_1_.

In the literature, the performance of adaptation is usually characterized by two quantities: the adaptation sensitivity and accuracy [3, 5]. Let *ϕ*_*peak*_ denote the peak value of the output *ϕ*(*t*). Then the adaptation sensitivity *γ* and the adaptation error *ϵ* are defined respectively by

$$\begin{array}{c} \gamma =\frac{\left(\phi_{peak}-\phi_{0}\right)/\phi_{0}}{\left(I_{1}-I_{0}\right)/I_{0}},\;\;\;\epsilon =\frac{\left(\phi_{\infty }-\phi_{0}\right)/\phi_{0}}{\left(I_{1}-I_{0}\right)/I_{0}}. \end{array}$$

The adaptation accuracy *δ* is then defined as the inverse of the adaptation error, namely, *δ* = 1/*ϵ*. In realistic biochemical systems, we hope the sensitivity *γ* and the accuracy *δ* to be as large as possible. In this way, the systems could detect the environmental fluctuations more sensitively and recover to their initial states more accurately.

In this paper, we define another indicator named as adaptation efficiency that can reflect the information of both the sensitivity *γ* and the accuracy *δ*. The adaptation efficiency *η* of the system is defined by

$$\begin{array}{c} \eta =1-\frac{\epsilon }{\gamma }=1-\frac{1}{\gamma \delta }=\frac{\phi_{peak}-\phi_{\infty }}{\phi_{peak}-\phi_{0}}, \end{array}$$ (3)

which is a number between 0 and 1. It is obvious that with the increase of *γ* and *δ*, the efficiency *η* will become larger. Thus in realistic biochemical systems, we hope the efficiency *η* to be as large as possible.

In general, an adaptation system will perform overshoot. Unlike the definitions of *γ* and *ϵ*, the definition of the efficiency *η* does not depend on the input *I*. This shows that the concept of efficiency can also be introduced for overshoot.

### Sensory network model of adaptation in *E. coli*

As a paradigmatic example of adaptation systems, the *E. coli* chemotactic sensory system has been studied extensively over the years [6–8, 31, 34, 39]. Here we briefly recall a popular model of adaptation in *E. coli* to help the readers understand the basic framework introduced above.

The core component of the *E. coli* chemotactic sensory system is the transmembrane methyl-accepting chemotaxis protein (MCP) receptor. MCP receptors bind selectively to the ligands outside the plasma membrane and modulate the activity of its downstream signal transduction pathway. Specifically, the activity of MCP receptors regulates the clockwise-counterclockwise rotational switch of downstream flagellar motors, which drive the run-and-tumble motion of an *E. coli* cell.

**Fig 2:**
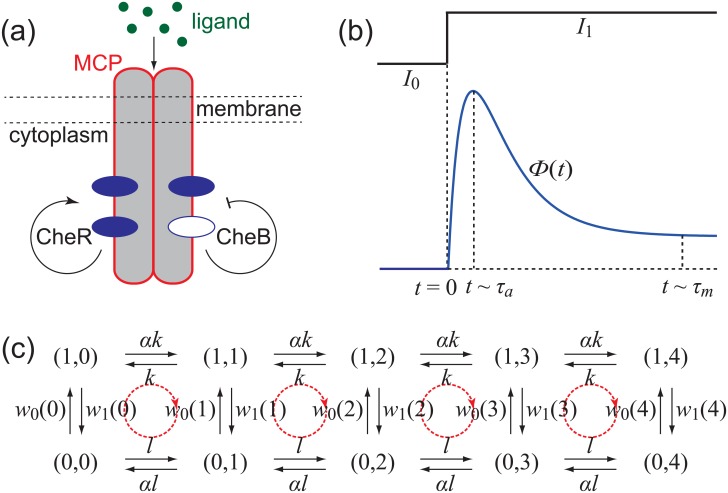
The *E. coli* chemotactic sensory system. (a) A schematic diagram of the MCP receptor. (b) The response of the MCP receptor to a step increase of the ligand concentration. (c) The sensory network model of the MCP receptor.

A schematic diagram of an MCP receptor is illustrated in Fig 2(a). The activity of the receptor can be described by a binary variable *a*, where *a* = 1 represents the active state and *a* = 0 represents the inactive state. Moreover, each MCP receptor has four methylation sites and thus the methylation level of the receptor can be represented by a variable *m*, where *m* ranges from 0 to 4. The change of the methylation level *m* relies on methylation and demethylation of the receptor by the enzymes CheR and CheB, respectively.

Recently, an influential model of the MCP receptor called the sensory network model has been proposed by Lan et al [5]. In their model, each state can be represented as (*a*, *m*), where *a* is the activity and *m* is the methylation level (see Fig 2(c)). The transitions between the active and inactive states take place on the time scale *τ*_*a*_ in a way that depends on the methylation level *m* and the ligand concentration *I*, with rates given by

$$\begin{array}{c} w_{1}\left(m\right)=\frac{1}{\tau_{a}}\exp\left(\frac{E(m,I)}{2k_{B}T}\right),\;\;\;w_{0}\left(m\right)=\frac{1}{\tau_{a}}\exp\left(-\frac{E(m,I)}{2k_{B}T}\right), \end{array}$$

where *k*_*B*_ is Boltzmann’s constant, *T* is the temperature, and *E*(*m*, *I*) = *e*_0_(*m*_0_ − *m*) + *f*(*I*) is the free energy difference between states (0,*m*) and (1,*m*). Here *e*_0_ is the methylation energy, *m*_0_ is an offset methylation level, and *f*(*I*) = *k*_*B*_ *T* log[(1 + *I*/*K*_*i*_)/(1+*I*/*K*_*a*_)] gives the chemical potential difference between the active and inactive states due to the binding of ligands with respective equilibrium constants *K*_*a*_ and *K*_*i*_ [5]. The transitions between different methylation levels take place on the time scale *τ*_*m*_ in a way that depends on the activity of the receptor. When the receptor is active, the rate of methylation is *αk* while the rate of demethylation is *k*. When the receptor is inactive, the rate of demethylation is *αl* while the rate of methylation is *l*. The methylation-demethylation cycle for the MCP receptor is fueled by the hydrolysis of SAM which donates a methyl group to an inactive receptor. The effect of the energy supply on the methylation and demethylation rates is represented by the parameter *α*, whose biophysical origin is discussed in [5, 29].

The output of the sensory network model is usually chosen as the sum of probabilities of the five inactive states:

$$\begin{array}{c} \phi \left(t\right)=\sum_{m=0}^{4}p_{\left(0,m\right)}\left(t\right). \end{array}$$ (4)

In fact, 1 − *ϕ*(*t*) exactly describes the mean activity of the system. The response of the MCP receptor to a step increase of the ligand concentration obtained from experimental observations is illustrated in Fig 2(b), from which we see that the system performs adaptation. The output *ϕ*(*t*) changes sharply on a very short time scale *τ*_*a*_ less than a second, and then recovers slowly on a much longer time scale *τ*_*m*_ with the order of a minute [5, 34]. Thus *τ*_*a*_ and *τ*_*m*_ characterize the time scales of the response and relaxation processes, respectively.

### Simple overshoot and oscillatory overshoot

Recently, it has been shown that overshoot can be classified into two different types: simple overshoot and oscillatory overshoot [30]. Here we briefly recall these two concepts. If the system has only two states, then the output has the form of *ϕ*(*t*) = *ϕ*_∞_+*c*_1_*e*^*λ*_1_*t*^. In this case, the output *ϕ*(*t*) must be a monotonic function and thus the system can never perform overshoot.

The situation is totally different in systems with three or more states. For simplicity, we consider a three-state system. From Eq (2), the output *ϕ*(*t*) of a three-state system is given by

$$\begin{array}{c} \phi \left(t\right)=\phi_{\infty }+c_{1}e^{\lambda_{1}t}+c_{2}e^{\lambda_{2}t}, \end{array}$$ (5)

where *λ*_1_ and *λ*_2_ are the nonzero eigenvalues of the matrix *Q*. There are two different situations: either *λ*_1_ and *λ*_2_ are both negative real numbers, or *λ*_1_ and *λ*_2_ are conjugate complex numbers with a negative real part.

**Fig 3:**
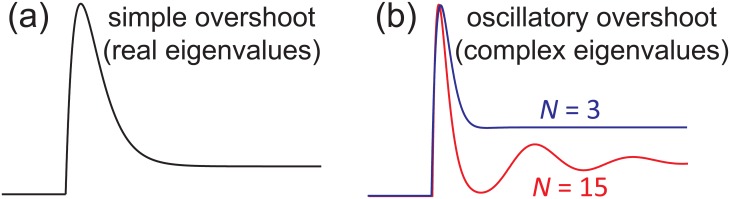
Simple overshoot and oscillatory overshoot. (a) Simple overshoot. (b) Oscillatory overshoot. When the number of states is small, the oscillation of the output can hardly be observed. When the number of states is large, the oscillation of the output may become apparent.

If *λ*_1_ and *λ*_2_ are negative real numbers and the signs of *c*_1_ and *c*_2_ are not the same, then *ϕ*(*t*) may not be a monotonic function and thus the system may perform overshoot. In this case, the output first rises to a peak and then declines directly toward a lower plateau. This type of overshoot is referred to as simple overshoot (see Fig 3(a)).

If *λ*_1_ and *λ*_2_ are conjugate complex numbers with a negative real part, we may denote them as λ_1_ = −λ + *ω*i and λ_2_ = −λ − *ω*i, where *λ* is a positive real number. With these notations, the output *ϕ*(*t*) can be rewritten as

$$\begin{array}{c} \phi \left(t\right)=\phi_{\infty }+2\left|c_{1}\right|e^{-\lambda t}\cos\left(\omega t+\varphi \right), \end{array}$$

where *φ* is the argument of *c*_1_. In this case, after the output rises to a peak and declines toward its initial value, it may rise and decline again to form a damped oscillation with decaying rate *λ* and angular frequency *ω*. This type of overshoot is called oscillatory overshoot.

Similarly, we can also classify adaptation into two different types: simple adaptation and oscillatory adaptation, whose essential difference is whether the system exhibits an oscillatory response.

## Results

### Reason why oscillatory adaptation is seldom reported

We have seen that adaptation can be classified into two different types, according to whether the system exhibits an oscillatory response. In biochemical systems modeled by continuous Markov models such as stochastic differential equations, the oscillation of the output is widely observed and this phenomenon is referred to as stochastic oscillation [40, 41]. In biochemical systems modeled by discrete Markov models, however, the vast majority of documented cases are adaptation without oscillatory dynamics, whereas adaptation with oscillatory dynamics is rarely reported.

**Fig 4:**
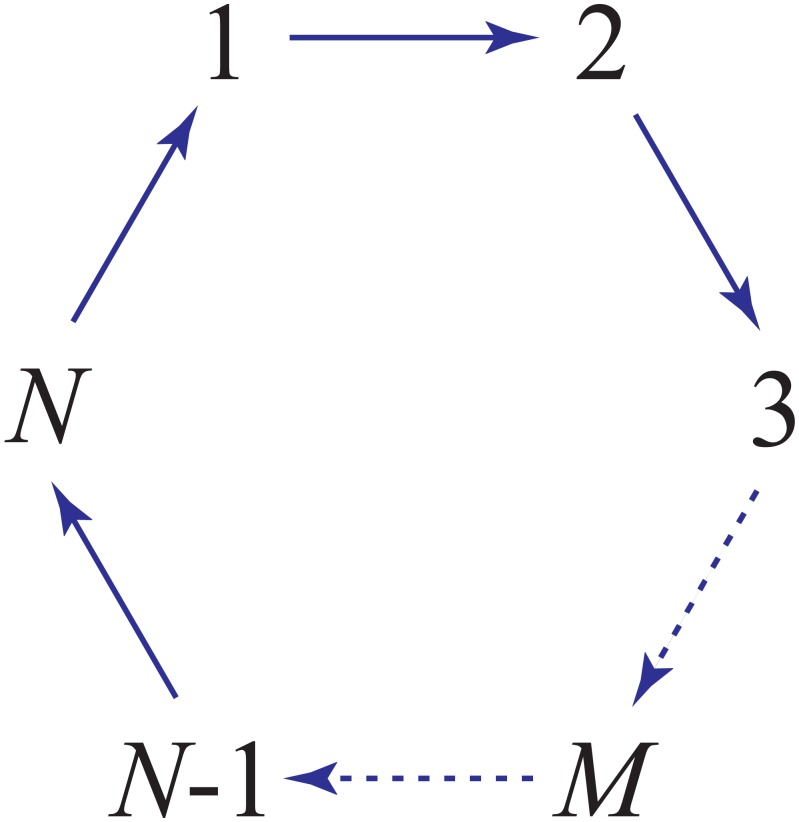
The sufficient and necessary conditions such that the equality in Eq (6) holds. The system is required to have a cyclic topological structure. Moreover, the transition rates along the forward cycle (blue arrows) need to be the same and the transition rates along the backward cycle need to be all zero.

To explain this phenomenon, we need a mathematical result established by Dmitriev and Dynkin [42] and Karpelevich [43] about the eigenvalues of generator matrices. The Dmitriev-Dynkin-Karpelevich theorem claims that if a Markov system has *N* different states, then any pair of conjugate complex eigenvalues −λ ± *ω*i of the generator matrix *Q* must satisfy

$$\begin{array}{c} \frac{\lambda }{\left|\omega \right|}\geq \tan\frac{\pi }{N}, \end{array}$$ (6)

where the equality holds if and only if the following three conditions are satisfied (see Fig 4):

1. the system has a cyclic topological structure;
2. the transition rates along the forward cycle are the same;
3. the transition rates along the back cycle are all zero.

In particular, if a Markov system has only three states, the above inequality reduces to $\lambda /\left|\omega \right|\geq \sqrt{3}$. This suggests that in a three-state system, the decaying rate *λ* of the output must be very fast compared to the angular frequency *ω*. Before the output could enter the second period of the oscillation, its amplitude has decayed exponentially to a neighborhood of its steady-state value that is too small to be observed, as illustrated by the blue curve in Fig 3(b).

The situation is somewhat different when the system has a large number of states, in which case the term tan(*π*/*N*) becomes very small. If *λ*/|*ω*| ≈ tan(*π*/*N*), then the decaying rate *λ* will be very slow compared to the angular frequency *ω*. In this case, the output may oscillate many times before approaching its steady-state value, as illustrated by the red curve in Fig 3(b). However, if *λ*/|*ω*| deviates significantly from tan(*π*/*N*), then the decaying rate *λ* will be very fast compared to the angular frequency *ω*. In this case, the oscillation of the output is hard to be observed.

In realistic biochemical systems, the number of states is usually not very large. Even if a biochemical system has a large number of states, the three conditions below Eq (6) are difficult to be satisfied, which implies that *λ*/|*ω*| cannot be very small. Therefore, in practice, it is difficult to distinguish oscillatory adaptation from simple adaptation by the experimentally observed output curve. Even if a biochemical system performs oscillatory adaptation, the oscillation of the output can hardly be observed. This explains why adaptation with oscillatory dynamics is rarely reported.

### Phase transition associated with overshoot

If the surrounding conditions remain constant, a biochemical system will reach a steady state for which two classes must be distinguished. In an equilibrium state, the detailed balance condition *μ*_*i*_ *q*_*ij*_ = *μ*_*j*_ *q*_*ji*_ holds for any pair of states *i* and *j* and there are no net fluxes between adjacent states. In an NESS, however, the detailed balance condition is broken. The nonzero net fluxes between adjacent states indicate that the system is externally driven with concomitant energy dissipation [32, 44]. Natural biochemical systems are highly dissipative, consuming energy to carry out various important biological functions. If there is a sustained energy supply from the environment, then the system will reach an NESS.

The central aim of this paper is to study the effects of nonequilibrium on adaptation. To this end, we only need to study the relationship between overshoot and the net flux. For simplicity, we consider a three-state system. The steady-state distribution *μ* = (*μ*_1_, *μ*_2_, *μ*_3_) of a three-state system must satisfy *μQ* = 0, which is equivalent to saying that

$$\begin{array}{c} \mu_{1}q_{12}-\mu_{2}q_{21}=\mu_{2}q_{23}-\mu_{3}q_{32}=\mu_{3}q_{31}-\mu_{1}q_{13}=J, \end{array}$$ (7)

where *J* is referred to as the net flux of the system. It is easy to see that the system satisfies detailed balance if and only if *J* = 0. Thus the net flux *J* is an effective indicator that describes the strength of the nonequilibrium driving. In this paper, we only consider the case of *J* ≥ 0. The case of *J* ≤ 0 can be analyzed in a similar way.

For convenience, we introduce the following three parameters:

$$\begin{array}{c} a=\mu_{1}q_{12}+\mu_{2}q_{21},\;\;\;b=\mu_{2}q_{23}+\mu_{3}q_{32},\;\;\;c=\mu_{3}q_{31}+\mu_{1}q_{13}. \end{array}$$

With these parameters, the generator matrix *Q* can be represented by the steady-state distribution *μ* and the net flux *J* as

$$\begin{array}{c} Q=\left[\begin{array}{ccc} -\left(c+a\right)/2\mu_{1} & \left(a+J\right)/2\mu_{1} & \left(c-J\right)/2\mu_{1}\\ \left(a-J\right)/2\mu_{2} & -\left(a+b\right)/2\mu_{2} & \left(b+J\right)/2\mu_{2}\\ \left(c+J\right)/2\mu_{3} & \left(b-J\right)/2\mu_{3} & -\left(b+c\right)/2\mu_{3} \end{array}\right]. \end{array}$$ (8)

Let *λ*_1_ and *λ*_2_ denote the two nonzero eigenvalues of *Q*. Direct computation shows that *λ*_1_ and *λ*_2_ are conjugate complex numbers if and only if *J* > *β* (see S1 Appendix:2), where *β* is a nonnegative constant defined as

$$\begin{array}{c} \beta =\sqrt{\frac{\mu_{1}\mu_{2}\mu_{3}}{4}{\left(\frac{c+a}{\mu_{1}}+\frac{a+b}{\mu_{2}}+\frac{b+c}{\mu_{3}}\right)}^{2}-\left(ab+bc+ca\right)}. \end{array}$$

This shows that *β* is a critical value of the net flux. If *J* > *β*, then *λ*_1_ and *λ*_2_ are conjugate complex numbers and the system will perform oscillating overshoot, whereas if *J* ≤ *β*, then *λ*_1_ and *λ*_2_ are real numbers and oscillating overshoot will not occur.

In the case of *J* ≤ *β*, the system may perform non-overshoot or simple overshoot. Without loss of generality, we assume that *λ*_1_ ≥ *λ*_2_. For convenience, we introduce the following two parameters:

$$\begin{array}{c} E=\phi^{\prime }\left(0\right),\;\;\;F=\phi_{\infty }-\phi_{0}. \end{array}$$

It can be proved that when *J* ≤ *β*, the system performs simple overshoot if and only if *E* + *λ*_2_ *F* > 0 (see S1 Appendix:3).

**Fig 5:**
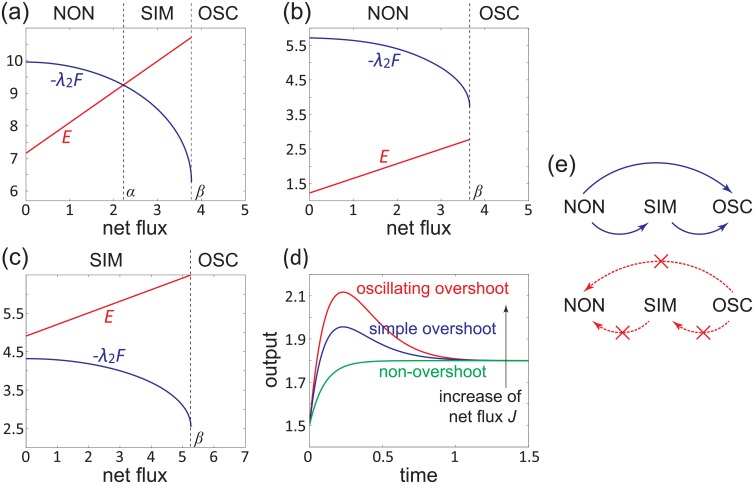
Three types of phase transitions as the net flux varies. (a)-(c) The dependence of *E* (red line) and −*λ*_2_ *F* (blue line) on the net flux *J*. (a) The red and blue lines intersect at *J* = *α*. (b) The red line is below the blue line. (c) The red line is above the blue line. (d) The change of the output as the net flux increases. (e) The possible directions (blue arrows) and impossible directions (red arrows) of the phase transition as the nonequilibrium driving becomes stronger.

In order to study the effects of the net flux on simple overshoot, we only need to study the dependence of *E* and −*λ*_2_ *F* on *J* and compare which one of *E* and −*λ*_2_ *F* is larger. Direct computation shows that

$$\begin{array}{c} E=a_{1}+a_{2}J,\;\;\;-\lambda_{2}F=a_{3}+a_{4}\sqrt{\beta^{2}-J^{2}}, \end{array}$$

where *a*_1_, *a*_2_, *a*_3_, and *a*_4_ are nonnegative constants independent of *J* (see S1 Appendix:4). This suggests that *E* is an increasing function of *J* and −*λ*_2_ *F* is a decreasing function of *J*. As *J* varies between 0 and *β*, the dependence of *E* and −*λ*_2_ *F* on *J* is illustrated by the red and blue lines in Fig 5(a)–5(c), respectively. The system performs simple overshoot if and only if the red line is above the blue line.

We have seen that a biochemical system may perform three different types of dynamic behavior: non-overshoot (NON), simple overshoot (SIM), and oscillatory overshoot (OSC). With the increase of *J*, the system may undergo three different types of phase transitions which are listed below.

The first type of phase transition is illustrated in Fig 5(a), where the red and blue lines intersect at *J* = *α*. This shows that the net flux has two critical values *α* and *β*. At the critical value *α*, the system undergoes a phase transition between non-overshoot and simple overshoot. At the critical value *β*, the system undergoes a phase transition between simple overshoot and oscillatory overshoot. With the increase of *J*, all the three types of dynamic behavior will appear (see Fig 5(d)).

The second type of phase transition is illustrated in Fig 5(b), where the red line is below the blue line. This type of phase transition can be viewed as the degenerate case of *α* = *β*. At the critical value *β*, the system undergoes a phase transition between non-overshoot and oscillatory overshoot. In this case, *J* cannot vary between *α* and *β* and thus simple overshoot will not appear.

The third type of phase transition is illustrated in Fig 5(c), where the red line is above the blue line. This type of phase transition can be viewed as the degenerate case of *α* = 0. At the critical value *β*, the system undergoes a phase transition between simple overshoot and oscillatory overshoot. In this case, *J* cannot vary between 0 and *α* and thus non-overshoot will not appear.

In summary, we have seen that the net flux has two critical values *α* and *β*. When 0 ≤ *J* ≤ *α*, the system will not perform overshoot; when *α* < *J* ≤ *β*, the system will perform simple overshoot; when *J* > *β*, the system will perform oscillatory overshoot.

### Overshoot and nonequilibrium

We shall next discuss the relationship between overshoot and nonequilibrium. If a system is in equilibrium, then the detailed balance condition *μ*_*i*_ *q*_*ij*_ = *μ*_*j*_ *q*_*ji*_ holds for any pair of states *i* and *j*. Let $M=\text{diag}(\sqrt{\mu_{1}},\cdots ,\sqrt{\mu_{N}})$ denote the diagonal matrix whose diagonal entries are $\sqrt{\mu_{1}},\cdots ,\sqrt{\mu_{N}}$, respectively. Then the detailed balance condition suggests that the matrix

$$\begin{array}{c} S=MQM^{-1}=\left(\frac{\sqrt{\mu_{i}}q_{ij}}{\sqrt{\mu_{j}}}\right) \end{array}$$

is symmetric. It is an elementary result in linear algebra that the eigenvalues of a symmetric matrix must be all real numbers. Since *Q* and *S* only differ up to a similar transform, they must have the same eigenvalues. This shows if a system is in equilibrium, then the eigenvalues of the generator matrix *Q* must be all real numbers. As a result, oscillatory overshoot can never occur in equilibrium systems.

Unfortunately, a similar result does not hold for simple overshoot. For the third type of phase transition illustrated in Fig 5(c), the system will perform simple overshoot even when the net flux vanishes. This indicates that simple overshoot may occur in equilibrium systems, which is consistent with the early work that used equilibrium Markov models to describe adaptation in various sensory systems [17, 21–27].

Despite all this, there is still a close relationship between overshoot and nonequilibrium. We make a crucial observation that for all the three types of phase transitions illustrated in Fig 5(a)–5(c), with the increase of the net flux, the system will undergo a phase transition along a fixed direction: from non-overshoot to simple overshoot then to oscillatory overshoot, although a particular phase may be skipped during the phase transition. The possible directions of the phase transition are illustrated by the blue arrows in Fig 5(e). In addition, the phase transition in the opposite direction is forbidden. Specifically, as the nonequilibrium driving becomes stronger, the phase transition from oscillatory overshoot to simple overshoot, that from oscillatory overshoot to non-overshoot, and that from simple overshoot to non-overshoot are all impossible, as illustrated by the red arrows in Fig 5(e). All these results indicate that overshoot and adaptation tend to occur in systems far away from equilibrium. Therefore, in realistic biochemical systems, adaptation is likely a good indicator of nonequilibrium dynamics.

### Nonequilibrium enhances adaptation efficiency

To gain a deeper insight into the relationship between adaptation and nonequilibrium, we shall study the effects of the net flux on the adaptation efficiency. For simplicity, we consider a three-state system and continue to use the parametrization Eq (8) of the generator matrix *Q*.

In order to obtain an explicit dependence of the adaptation efficiency *η* on the net flux *J*, we need to make an additional assumption. We assume that state 1 has relatively fast transitions compared to the other two states. Under this assumption, the state transitions of the system have two separate time scales. In realistic sensory systems, adaptation always possesses a multiscale feature: in response to an abrupt input change, the output usually changes sharply on a very short time scale and recovers slowly over a much longer time scale. In this way, the system could detect the environmental fluctuations more rapidly. Thus the above assumption of time scale separation does not impose too much restriction onto the system.

**Fig 6:**
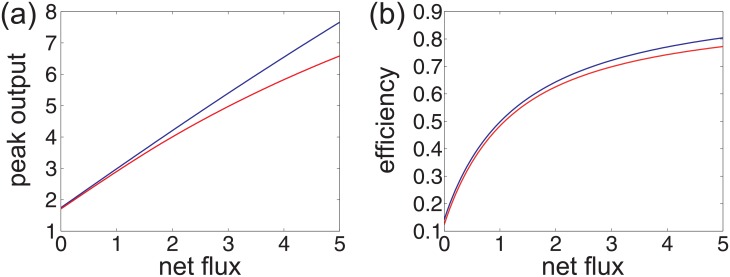
Nonequilibrium enhances the adaptation efficiency. The red lines illustrate the case where the state transitions of the system do not have a separate time scale. In this case, the model parameters are taken as *π* = (1, 0, 0), *μ* = (0.1, 0.5, 0.4), *g* = (0, 2, 1), *a* = 1.2, *b* = 0.6, and *c* = 0.2. The blue lines illustrate the case where state 1 has relatively fast transitions compared to the other two states. In this case, all the model parameters remain the same except that we take *μ* = (0.0001, 0.5, 0.4999). (a) The peak output versus the net flux. (b) The adaptation efficiency versus the net flux.

If state 1 has relatively fast transitions compared to the other two states, then the process with the fast time scale can be eliminated [45, 46]. Recall that the system will perform overshoot provided *J* > *α*. In the case of *J* > *α*, it can be proved that the peak output *ϕ*_*peak*_ is a approximate linear function of *J*, namely,

$$\begin{array}{c} \phi_{peak}\approx b_{1}+b_{2}J, \end{array}$$

where *b*_1_ and *b*_2_ are two nonnegative constants independent of *J* (see S1 Appendix:5), as illustrated by the blue line in Fig 6(a). With the increase of *J*, the initial output *ϕ*_0_ and the steady-state output *ϕ*_∞_ remain invariant, whereas the peak output *ϕ*_*peak*_ will become larger (see Fig 5(d)). From Eq (3), the adaptation efficiency *η* can be rewritten as

$$\begin{array}{c} \eta \approx 1-\frac{\phi_{\infty }-\phi_{0}}{b_{1}+b_{2}J-\phi_{0}}. \end{array}$$

This suggests that the adaptation efficiency will also increase as the nonequilibrium driving becomes stronger (see Fig 6(b)).

The readers may ask what will happen if the state transitions of the system do not have two separate time scales. In this case, we cannot discard the fast process and it is difficult to obtain an explicit dependence of the peak output on the net flux. However, according to our numerical computation, we find that similar results still hold. The numerical results show that *ϕ*_*peak*_ is still an increasing function of *J*, but no longer depends on *J* linearly, as illustrated by the red line in Fig 6(a). From Eq (3), it is easy to see that the adaptation efficiency *η* is also an increasing function of *J* (see Fig 6(b)). This indicates that nonequilibrium will always enhance the adaptation efficiency whether the system has two separate time scales or not.

### Phase transition in the sensory network model

In the above discussions, we mainly develop our theory in simple Markov models. A natural question is whether our main results also hold in realistic biochemical systems with relatively complex networks. Here we shall validate the main results of this paper by studying the relationship between adaptation and nonequilibrium based on the sensory network model of the *E. coli* chemotactic sensory system (see Fig 2(c)).

It is well-known that whether a Markov system will reach an equilibrium state or an NESS is closely related to the cycles of the system. Recall that a Markov system satisfies the detailed balance condition if and only if the product of transition rates along any cycle and that along its reversed cycle are exactly the same [32]. In fact, the sensory network model has four basic cycles, as illustrated by the red arrows in Fig 2(c). By using the above criteria, it is easy to check that the sensory network model satisfies the detailed balance condition if and only if *α* = *e*^*e*_0_/2^. If *α* < *e*^*e*_0_/2^, the system is driven out of equilibrium with energy dissipation. Thus *α* is an effective parameter that characterizes the strength of the nonequilibrium driving.

We next discuss the effects of nonequilibrium on adaptation in the sensory network model using both analytic and numerical methods. In the following numerical computations, we adopt the model parameters as suggested in [5]: *e*_0_ = 2, *m*_0_ = 1, *K*_*i*_ = 18.2 *μ*M, *K*_*a*_ = 3000 *μ*M, and *k*_*B*_ *T* = 1. The ligand concentration is elevated from *I*_0_ = 10*K*_*i*_ to *I*_1_ = 15*K*_*i*_. The time constants are taken as *τ*_*a*_ = 0.1 s and *k* = *l* = 0.01 s^−1^. With this set of model parameters, an estimate of the typical methylation-demethylation cycle time is given by *τ*_*m*_ ≈ 1/*k* + 1/*l* = 200 s.

**Fig 7:**
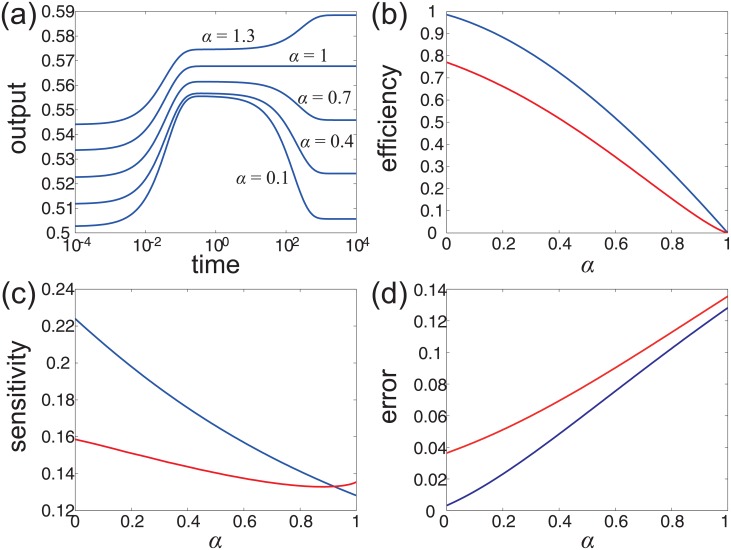
The relationship between adaptation and nonequilibrium in the sensory network model. (a) The response of the MCP receptor to a step increase of the ligand concentration under different choices of *α*. (b) The dependence of the adaptation efficiency on *α* under *τ*_*a*_ = 0.1 s (blue line) and *τ*_*a*_ = 200 s (red line). (c) The dependence of the adaptation sensitivity on *α* under *τ*_*a*_ = 0.1 s (blue line) and *τ*_*a*_ = 200 s (red line). (d) The dependence of the adaptation error on *α* under *τ*_*a*_ = 0.1 s (blue line) and *τ*_*a*_ = 200 s (red line).

The sensory network model has some nice mathematical properties. In fact, it can be proved that if the system satisfies the detailed balance condition, then adaptation will never occur, regardless of how the model parameters are chosen (see S1 Appendix:6). This indicates that nonequilibrium is a necessary condition for adaptation in the sensory network model. Moreover, with the model parameters taken above, it has been shown that the system performs adaptation if and only if *α* < 1 [31]. This shows that *α* = 1 is a critical value of adaptation. If 1 ≤ *α* ≤ *e*^*e*_0_/2^, the system will not perform adaptation, whereas if *α* < 1, the system will perform adaptation (see Fig 7(a)). According to our numerical computation, the system will undergo a phase transition between non-adaptation and simple adaptation at the critical value *α* = 1. If we further decrease *α*, the system still performs adaptation and the eigenvalues of the generator matrix are all real numbers. This shows that with the decrease of *α*, the system cannot further transition from simple adaptation to oscillatory adaptation.

The reason why oscillatory adaptation does not appear during the phase transition can also be explained by our theory. In fact, the conditions (b) and (c) below Eq (6) show that if we hope a Markov system to exhibit an apparent oscillatory response, the transition rates along any cycle cannot differ to much. In the sensory network model, all the vertical transition rates have the time scale of *τ*_*a*_ and all the horizontal ones have the time scale of *τ*_*m*_. Thus in order to make the sensory network model perform oscillatory adaptation, *τ*_*a*_ and *τ*_*m*_ cannot differ too much. To validate this claim, we change the time constant *τ*_*a*_ from 0.1 s to 200 s to ensure *τ*_*a*_ ≈ *τ*_*m*_. With this parameter value, *α* = 1 is still a critical value that distinguishes non-adaptation from simple adaptation, according to our numerical computation. If we further decrease *α*, then the system undergoes a phase transition from simple adaptation to oscillatory adaptation at another critical value *α* ≈ 0.91. With the decrease of *α*, the number of complex eigenvalues of the generator matrix increases, as shown in Table 1.

**Table 1:** The number of complex eigenvalues of the generator matrix under different ranges of *α*.

range of *α*	0–0.46	0.47–0.87	0.88–0.91	0.92–1
number of complex eigenvalues	6	4	2	0

All these numerical results indicate that as the nonequilibrium driving becomes stronger, the sensory network model will undergo a phase transition along a fixed direction: from non-adaptation to simple adaptation then to oscillatory adaptation, which coincides perfectly with our previous conclusion.

### Nonequilibrium enhances adaptation performance in the sensory network model

We have seen that in simple Markov models, nonequilibrium will enhance the adaptation efficiency. Here we shall study the effects of nonequilibrium on the performance of adaptation based on the sensory network model.

With the set of model parameters listed above, the dependence of the adaptation efficiency *η*, the adaptation sensitivity *γ*, and the adaptation error *ϵ* on the parameter *α* is illustrated by the blue lines in Fig 7(b)–7(d), respectively. With the decrease of *α*, the adaptation efficiency and sensitivity will increase and the adaptation error will decrease. This suggests that a strong nonequilibrium driving will make the system respond to the environmental fluctuations more sensitively and recover to the initial state more accurately. In other words, nonequilibrium will improve the performance of adaptation by enhancing the adaptation efficiency, sensitivity, and accuracy. This also coincides perfectly with our previous conclusion.

We next discuss another interesting topic. We have seen that the time constants *τ*_*a*_ and *τ*_*m*_ give the time scales of the response and relaxation processes, respectively (see Fig 2(b)). In realistic systems, *τ*_*a*_ is usually far less than *τ*_*m*_. In this way, the system possesses a fast response speed and a much slower adaptation speed. Interestingly, we find that the small time constant *τ*_*a*_ not only guarantees a fast response speed, but also improves the performance of adaptation.

To see this point, we change the time constant *τ*_*a*_ from 0.1 s to 200 s to ensure *τ*_*a*_ ≈ *τ*_*m*_. With this parameter value, the dependence of the efficiency *η*, the sensitivity *γ*, and the error *ϵ* on the parameter *α* is illustrated by the red lines in Fig 7(b)–7(d), respectively. By comparing the blue and red lines in Fig 7(b)–7(d), we can see that a small time constant *τ*_*a*_ will lead to a larger efficiency, a larger sensitivity, and a smaller error. When *τ*_*a*_ ≪ *τ*_*m*_, the efficiency approaches 1 as *α* tends to 0. However, when *τ*_*a*_ ≈ *τ*_*m*_, the efficiency has a maximum value less than 0.8. All these numerical results indicate that a fast transition between the active and inactive states not only enhances the response speed, but also enhances the adaptation efficiency, sensitivity, and accuracy.

## Conclusions and Discussions

The relationship between adaptation and nonequilibrium has gained increasing attention in recent years [5, 28–31]. In our previous work, we found that in some particular Markov models, nonequilibrium is a necessary condition for adaptation [30]. However, this result is not quite satisfactory. In fact, it has been shown that some influential equilibrium models, such as the four-state model based on ligand-induced receptor modification proposed by Segel, Goldbeter, and coworkers, can achieve accurate adaptation [21–24]. In addition, Ao et al [28] found that a three-state equilibrium model may perform overshoot. These results indicate that even in a simple Markov model, nonequilibrium is not always a necessary condition for adaptation. This appears to contradict recent studies, which emphasize the nonequilibrium effects of adaptation. How to understand these seemingly conflicting statements is the starting point of this paper.

In the present paper, we provide an explanation of this inconsistency by studying the effects of nonequilibrium on adaptation in stochastic biochemical systems. In simple Markov models, we reveal that as the nonequilibrium driving becomes stronger, the system will undergo a phase transition along a fixed direction: from non-adaptation to simple adaptation then to oscillatory adaptation, although a particular type of dynamic behavior may be skipped during the phase transition. If the non-adaptation phase is skipped, then the system will transition directly from simple adaptation to oscillatory adaptation. This shows that although adaptation may occur in equilibrium systems, it tends to occur in systems far from equilibrium. All these results provide a relatively satisfactory answer to the underlying contradiction in the previous studies.

In simple Markov models, we impose very few constraints on the topological structure, transition rates, initial distribution, and observable of the system. In the recent work of Ao et al [28], the authors showed that the choice of the observable may affect the analysis of overshoot: some observables are not good enough to discuss overshoot, while other observables may be better. In this paper, we find that the choice of the observable could be rather arbitrary and will hardly affect the theoretical analysis and main results. The only requirements for the observable are to make sure *ϕ*′(0) > 0 and *ϕ*_∞_ ≥ *ϕ*_0_. This suggests that our conclusions should be rather general.

In the previous literature, the performance of adaptation is usually characterized by two quantities: the adaptation sensitivity and accuracy [3, 5]. In this paper, we define another indicator named as adaptation efficiency that contains the information of both the sensitivity and accuracy. The most important advantage of the adaptation efficiency is that its definition does not depend on the input stimulus. In realistic biochemical systems, the transition rates between states may depend on the input stimulus in many different ways. Since the dependence of the transition rates on the input stimulus does not have a general form, it is difficult to perform a theoretical analysis on the sensitivity and accuracy in general Markov models.

In recent years, the relationship between nonequilibrium and the adaptation accuracy has been discussed in some particular Markov models such as the sensory network model and a coarse-grained model of stochastic differential equations [5, 29, 31]. Among these studies, Lan et al [5] found a relation among the energy dissipation rate, adaptation speed, and adaptation accuracy, which they claimed to hold generally. However, we make an important observation that the definition of the accuracy only depends on the initial and steady-state outputs. In order to perform an analysis on the accuracy, we only need to study the steady-state properties of the system, which is a relatively easy task.

In this paper, we study the effects of nonequilibrium on the adaptation efficiency, whose definition depends on the peak value of the output. In order to perform an analysis on the efficiency, we need to study the dynamic properties of Markov models, which turns out to be a very difficult problem. The previous work focuses more on the relaxation process of adaptation, while the present paper focuses more on the response process of adaptation, namely, overshoot. In addition to the phase transition associated with overshoot, we also find that a strong nonequilibrium driving will help a biochemical system enhance its adaptation efficiency. The study on the connection between nonequilibrium and the dynamic properties of adaptation, such as the transient response of overshoot and the adaptation efficiency, is probably the main innovative point of this paper.

A limitation of the present paper is that we mainly perform our analysis in simple Markov models. This is because if we hope to study the dynamic behavior of adaptation, we need to obtain an explicit expression of the output and analyze its properties such as monotonicity. To this end, we also need to calculate all the eigenvalues of the generator matrix. All these procedures become extremely difficult to be implemented if the system has a large number of states. However, we believe that the main results of this paper might be general principles that also apply to Markov models with relatively complex networks.

To validate whether our main results apply to realistic biochemical systems, we discuss the relationship between adaptation and nonequilibrium based on the sensory network model of the *E. coli* chemotactic sensory system using both analytic and numerical methods. It turns out that the numerical computations based on the sensory network model coincide perfectly with the main conclusions of this paper. Moreover, we prove that nonequilibrium is a necessary condition for adaptation in the sensory network model. In addition, we also find an interesting fact that a fast transition between the active and inactive states not only enhances the response speed, but also improves the performance of adaptation.

In the past, people tended to focus on the steady-state phenomena in biochemical systems. Recently, increasing attention has been paid to the dynamic phenomena in biochemical systems such as adaptation and overshoot. So far, there have been extensive studies on the equilibrium state and the NESS and these studies have been developed into a beautiful mathematical theory [32]. However, the study on the dynamic behavior of stochastic systems turns out to be a very difficult problem due to the lack of effective mathematical tools.

In this paper, we use several mathematical tools to perform an analysis on the dynamic properties of adaptation. These mathematical tools include the matrix theory, the properties of the eigenvalues of generator matrices, the mathematical theory of NESSs, and the mathematical theory of multiscale methods. We hope that the techniques used in this paper could be applied to the study of other dynamic phenomena in deterministic and stochastic biochemical systems.

## Supporting Information

S1 Appendix Detailed calculations. This file provides some detailed calculations that help the readers understand the content of the main text.
(PDF)
