# Supplementary material for: Nonequilibrium Enhances Adaptation Efficiency of Stochastic Biochemical Systems
Source: PLoS One. 2016 May 19;11(5):e0155838. doi: 10.1371/journal.pone.0155838 (PMC4873145; doi:10.1371/journal.pone.0155838)
Supplement: S1 Appendix — This file provides some detailed calculations that help the readers understand the content of the main text. (PDF) [file pone.0155838.s001.pdf]

S1 Appendix

Chen Jia, Minping Qian

Contents

1 **Calculation of the output $\phi(t)$** 1

2 **Calculation of the critical value β** 2

3 **Sufficient and necessary condition for simple overshoot** 3

4 **Calculation of E and $-\lambda_2 F$** 4

5 **Calculation of the peak output ϕ_{peak}** 5

6 **Adaptation in the sensory network model** 6

1 Calculation of the output $\phi(t)$

Let us consider a biochemical system modeled by a Markov jump process with N states, denoted by $1, 2, \cdots, N$ and generator matrix $Q = (q_{ij})$, where q_{ij} with $i \neq j$ denotes the transition rate from state i to j and $q_{ii} = -\sum_{j\neq i} q_{ij}$. Let $p(t) = (p_1(t), \cdots, p_N(t))$ denote the probability distribution of the system. Then the dynamics of the system is governed by the master equation

$$\begin{cases} \dfrac{dp(t)}{dt} = p(t)Q, \\ p(0) = \pi, \end{cases} \tag{1}$$

where $\pi = (\pi_1, \cdots, \pi_N)$ is the initial distribution of the system.

Let $g = (g_1, \cdots, g_N)$ denote the observable of the system and we assume that $g_i \geq 0$ for simplicity. Recall that the output $\phi(t)$ of the system is defined as

$$\phi(t) = \sum_i p_i(t) g_i = p(t) g^T.$$

By solving the master equation (1), it is easy to see that the output $\phi(t)$ has the form of

$$\phi(t) = \pi e^{Qt} g^T. \tag{2}$$

Let $\lambda_1, \cdots, \lambda_N$ denote all the eigenvalues of Q. Since we have assumed that the matrix Q has N different eigenvalues, it can be diagonalized. Specifically, there exists a complex matrix L such that

$$Q = LDL^T, \tag{3}$$

where $D = \text{diag}(\lambda_1, \cdots, \lambda_N)$ is a diagonal matrix whose diagonal elements are $\lambda_1, \cdots, \lambda_N$, respectively. From (2) and (3), it is easy to see that the output $\phi(t)$ is nothing but a linear combination of exponential functions:

$$\phi(t) = \pi L e^{Dt} L^T g^T = \sum_i c_i e^{\lambda_i t}, \tag{4}$$

where $c_1, \cdots, c_N$ are N constants. The Perron-Frobenius theorem [1] in the matrix theory claims that one eigenvalue of Q must be zero and the real parts of other eigenvalues are all negative. In the sequel, we fix $\lambda_N = 0$. By taking $t \to \infty$ in (4), we obtain that

$$c_N = \phi_\infty,$$

where ϕ_∞ is the steady-state output of the system. Thus the output $\phi(t)$ can be rewritten as

$$\phi(t) = \phi_\infty + \sum_{i=1}^{N-1} c_i e^{\lambda_i t}. \tag{5}$$

Since the output $\phi(t)$ is a real function, we have $\phi(t) = \bar{\phi}(t)$. By taking complex conjugate in (5), we obtain that

$$\sum_{i=1}^{N-1} c_i e^{\lambda_i t} = \sum_{i=1}^{N-1} \bar{c}_i e^{\bar{\lambda}_i t}.$$

Since $\lambda_1, \cdots, \lambda_N$ are mutually different, $e^{\lambda_1 t}, \cdots, e^{\lambda_N t}$ are linearly independent functions. Thus if λ_i is a real number, then c_i is also a real number, whereas if λ_i and λ_j are conjugate complex numbers, then c_i and c_j are also conjugate complex numbers.

2 Calculation of the critical value β

In this section, let us consider a three-state system. Let $\mu = (\mu_1, \cdots, \mu_N)$ denote the steady-state distribution of the system. Then the net flux J of the system is defined as

$$J = \mu_1 q_{12} - \mu_2 q_{21} = \mu_2 q_{23} - \mu_3 q_{32} = \mu_3 q_{31} - \mu_1 q_{13}. \tag{6}$$

For simplicity, we only consider the case of $J \geq 0$. The case of $J \leq 0$ can be analyzed in a similar way.

For convenience, we introduce the following three parameters:

$$a = \mu_1 q_{12} + \mu_2 q_{21}, \quad b = \mu_2 q_{23} + \mu_3 q_{32}, \quad c = \mu_3 q_{31} + \mu_1 q_{13}.$$

With these notations, the generator matrix Q can be represented by the steady-state distribution μ and the net flux J as

$$Q = \begin{bmatrix} -(c+a)/2\mu_1 & (a+J)/2\mu_1 & (c-J)/2\mu_1 \\ (a-J)/2\mu_2 & -(a+b)/2\mu_2 & (b+J)/2\mu_2 \\ (c+J)/2\mu_3 & (b-J)/2\mu_3 & -(b+c)/2\mu_3 \end{bmatrix}. \tag{7}$$

Direct computation shows that the characteristic polynomial of Q is given by

$$\det(\lambda I - Q) = \lambda(\lambda^2 + u\lambda + v),$$

where

$$u = \frac{c+a}{2\mu_1} + \frac{a+b}{2\mu_2} + \frac{b+c}{2\mu_3}, \quad v = \frac{ab+bc+ca}{4\mu_1\mu_2\mu_3} + \frac{J^2}{4\mu_1\mu_2\mu_3}.$$

Let λ_1 and λ_2 denote the two nonzero eigenvalues of Q. Then λ_1 and λ_2 are the two solutions to the quadratic equation

$$\lambda^2 + u\lambda + v = 0. \tag{8}$$

Recall that the discriminant Δ of this quadratic equation is defined as $\Delta = u^2 - 4v$. If $\Delta \geq 0$, then λ_1 and λ_2 are real numbers, whereas if $\Delta < 0$, then λ_1 and λ_2 are conjugate complex numbers. Direct computation shows that

$$\Delta = u^2 - \frac{ab+bc+ca}{\mu_1\mu_2\mu_3} - \frac{J^2}{\mu_1\mu_2\mu_3}.$$

It is a classical result that if a system satisfies the detailed balance condition, then the eigenvalues of the generator matrix Q must be all real numbers [2]. From (6), it is easy to see that the system satisfies the detailed balance condition if and only if $J = 0$. The above two facts together show that $\Delta \geq 0$ when $J = 0$, which further implies that

$$\mu_1\mu_2\mu_3 u^2 - (ab+bc+ca) \geq 0.$$

Thus the discriminant Δ can be rewritten as

$$\Delta = \frac{1}{\mu_1\mu_2\mu_3}(\beta^2 - J^2).$$

where β is a nonnegative constant defined as

$$\beta = \sqrt{\mu_1\mu_2\mu_3 u^2 - (ab+bc+ca)}.$$

It is easy to see that $\Delta < 0$ if and only if $J > \beta$. This shows that β is a critical value of the net flux. If $J > \beta$, then λ_1 and λ_2 are conjugate complex numbers and the system will perform oscillating overshoot, whereas if $J \leq \beta$, then λ_1 and λ_2 are real numbers and oscillating overshoot will not occur.

3 Sufficient and necessary condition for simple overshoot

In this section, we shall study the relationship between simple overshoot and the net flux in a three-state system. It is easy to see that simple overshoot can only occur when $J \leq \beta$. In the case of $J \leq \beta$, we have seen that λ_1 and λ_2 are real numbers and we assume that $\lambda_1 \geq \lambda_2$.

For convenience, we introduce the following two parameters:

$$E = \phi'(0), \quad F = \phi_\infty - \phi_0,$$

where ϕ_0 is the initial output of the system. From (5), the output $\phi(t)$ of the system is given by

$$\phi(t) = \phi_\infty + c_1 e^{\lambda_1 t} + c_2 e^{\lambda_2 t}.$$

Thus the derivative of $\phi(t)$ is given by

$$\phi'(t) = \lambda_1 c_1 e^{\lambda_1 t} + \lambda_2 c_2 e^{\lambda_2 t}.$$

This shows that

$$\begin{cases} E = \phi'(0) = \lambda_1 c_1 + \lambda_2 c_2, \\ F = \phi_\infty - \phi_0 = -c_1 - c_2. \end{cases}$$

Solving this set of equations, we obtain that

$$c_1 = \frac{E + \lambda_2 F}{\lambda_1 - \lambda_2}, \quad c_2 = -\frac{E + \lambda_1 F}{\lambda_1 - \lambda_2}.$$

Thus the output $\phi(t)$ can be rewritten as

$$\phi(t) = \phi_\infty + \frac{E + \lambda_2 F}{\lambda_1 - \lambda_2} e^{\lambda_1 t} - \frac{E + \lambda_1 F}{\lambda_1 - \lambda_2} e^{\lambda_2 t}. \tag{9}$$

It is easy to see that when $J \leq \beta$, the system performs simple overshoot if and only if the output $\phi(t)$ is a non-monotonic function. The following theorem gives a sufficient and necessary condition for the output being non-monotonic.

Theorem 1. Assume that $J \leq \beta$. Then $\phi(t)$ is non-monotonic if and only if $E + \lambda_2 F > 0$.

Proof. Note that $\phi(t)$ is non-monotonic if and only if $\phi'(t)$ has a zero in $(0, \infty)$. It follows from (9) that

$$\phi'(t) = \left[\gamma e^{(\lambda_1 - \lambda_2)t} - (\gamma - E)\right] e^{\lambda_2 t},$$

where

$$\gamma = \frac{\lambda_1 (E + \lambda_2 F)}{\lambda_1 - \lambda_2}.$$

This suggests that $\phi'(t)$ has a zero in $(0, \infty)$ if and only if $\gamma < 0$. Recall that $\lambda_1 < 0$ and $\lambda_1 > \lambda_2$. This indicates that $\phi(t)$ is non-monotonic if and only if $E + \lambda_2 F > 0$. □

This above theorem shows that when $J \leq \beta$, the system performs simple overshoot if and only if $E + \lambda_2 F > 0$.

4 Calculation of E and $-\lambda_2 F$

In this section, we shall study the dependence of E and $-\lambda_2 F$ on the net flux J. From (2), it is easy to check that

$$E = \phi'(0) = \pi Q g^T = a_1 + a_2 J,$$

where

$$a_1 = \left(\frac{\pi_2}{2\mu_2} - \frac{\pi_1}{2\mu_1}\right)(g_1 - g_2)a + \left(\frac{\pi_3}{2\mu_3} - \frac{\pi_2}{2\mu_2}\right)(g_2 - g_3)b + \left(\frac{\pi_1}{2\mu_1} - \frac{\pi_3}{2\mu_3}\right)(g_3 - g_1)c$$

and

$$a_2 = \frac{\pi_3}{2\mu_3}(g_1 - g_2) + \frac{\pi_1}{2\mu_1}(g_2 - g_3) + \frac{\pi_2}{2\mu_2}(g_3 - g_1). \tag{10}$$

Moreover, it is easy to check that

$$F = \phi_\infty - \phi_0 = \mu g^T - \pi g^T = (\mu_1 - \pi_1)g_1 + (\mu_2 - \pi_2)g_2 + (\mu_3 - \pi_3)g_3.$$

Recall that we always assume that $\phi'(0) > 0$ and $\phi_\infty \geq \phi_0$. In addition, we always focus on the case of $J \geq 0$. These two assumptions suggest that $F \geq 0$ and

$$E = a_1 + a_2 J > 0$$

for all $J \geq 0$. This implies that $a_1 > 0$ and $a_2 \geq 0$. Thus a_1 and a_2 are nonnegative constants independent of J.

By solving the quadratic equation (8), we obtain that

$$\lambda_1 = -\frac{u}{2} + \frac{1}{2}\sqrt{\frac{1}{\mu_1\mu_2\mu_3}(\beta^2 - J^2)},$$
$$\lambda_2 = -\frac{u}{2} - \frac{1}{2}\sqrt{\frac{1}{\mu_1\mu_2\mu_3}(\beta^2 - J^2)}.$$

This shows that

$$-\lambda_2 F = a_3 + a_4\sqrt{\beta^2 - J^2},$$

where

$$a_3 = \frac{uF}{2}, \quad a_4 = \frac{F}{2\sqrt{\mu_1\mu_2\mu_3}}.$$

Thus a_3 and a_4 are nonnegative constants independent of J.

5 Calculation of the peak output ϕ_{peak}

In this section, we shall study the dependence of the peak output ϕ_{peak} on the net flux J in a three-state system. To this end, we need to make an additional assumption. We assume that state 1 has relatively fast transitions compared to the other two states. Under this assumption, the state transitions of the system have two separate time scales.

In the main text, we have seen that the system performs overshoot if and only if $J > \alpha$. According to the mathematical theory of two-time-scale Markov chains [3, 4], it can be proved that when $J > \alpha$, the peak output ϕ_{peak} is approximately given by

$$\phi_{peak} \approx (\pi_2 + \frac{\pi_1 q_{12}}{q_{12} + q_{13}})g_2 + (\pi_3 + \frac{\pi_1 q_{13}}{q_{12} + q_{13}})g_3.$$

We continue to use the parametrization (7) of the generator matrix Q. With this parametrization, the peak output ϕ_{peak} can be rewritten as

$$\phi_{peak} \approx b_1 + b_2 J,$$

where

$$b_1 = (\pi_2 + \frac{\pi_1 a}{a+c})g_2 + (\pi_3 + \frac{\pi_1 c}{a+c})g_3, \quad b_2 = \frac{\pi_1(g_2 - g_3)}{a+c}.$$

Since state 1 has relatively fast transitions compared to the other two states, its steady-state probability must be much smaller than the steady-state probabilities of the other two states, namely, $\mu_1 \ll \mu_2$ and $\mu_1 \ll \mu_3$. In the above discussions, we have proved that $a_2 \geq 0$, where a_2 is the constant defined in (10). Under the assumption of $\mu_1 \ll \mu_2$ and $\mu_1 \ll \mu_3$, it is easy to see that the sign of a_2 is the same as that of $g_2 - g_3$. This shows that $g_2 - g_3 \geq 0$. Thus b_1 and b_2 are nonnegative constants independent of J.

6 Adaptation in the sensory network model

In this section, we shall prove that if the sensory network model satisfies the detailed balance condition, then it will never perform adaptation, regardless of how the model parameters are chosen. To prove this fact, we always assume that the system satisfies the detailed balance condition in the sequel.

Let I_0 and I_1 denote the initial and final input levels. When the input level is elevated from I_0 to I_1, the system is driven from a steady state to another one. The initial distribution π of the system is exactly the steady-state distribution for input I_0 and the final distribution μ is exactly the steady-state distribution for input I_1. Let $Q = (q_{ij})$ denote the generator matrix for input I_1 and let $\lambda_1, \cdots, \lambda_{10}$ denote all the eigenvalues of Q. It is a classical result that if a system satisfies the detailed balance condition, then the eigenvalues of the generator matrix Q must be all real numbers [2]. Without loss of generality, we assume that $\lambda_1 = 0$ and $\lambda_2, \cdots, \lambda_{10} < 0$.

For convenience, we shall relabel the states. The five inactivated states in the lower layer of the model are relabeled from state 1 to state 5 and the five activated states in the upper layer of the model are relabeled from state 6 to state 10. Let $M = \mathrm{diag}(\mu_1, \cdots, \mu_{10})$. Then the detailed balance condition $\mu_i q_{ij} = \mu_j q_{ji}$ implies that the matrix

$$S = M^{\frac{1}{2}} Q M^{-\frac{1}{2}} = \left(\frac{\sqrt{\mu_i} q_{ij}}{\sqrt{\mu_j}} \right)$$

is symmetric. Since S and Q only differ up to a similar transformation, they must have the same eigenvalues. According to the matrix theory, there exists an orthogonal matrix $R = (r_{ij})$ such that

$$RSR^T = D,$$

where $D = \mathrm{diag}(\lambda_1, \cdots, \lambda_{10})$. This suggests that the rows of the matrix R are exactly the unit eigenvectors of the matrix S. It is easy to check that the unit eigenvector of S associated with the eigenvalue $\lambda_1 = 0$ is $(\sqrt{\mu_1}, \cdots, \sqrt{\mu_{10}})$. Thus the orthogonality of R implies that

$$\sum_{k=1}^{10} \sqrt{\mu_k} r_{mk} = 0, \; m = 2, \cdots, 10. \tag{11}$$

By solving the master equation (1), it is easy to see that the probability $p_i(t)$ of state i is given by

$$p_i(t) = [\pi e^{tQ}]_i = [\pi M^{-\frac{1}{2}} R^{-1} e^{tD} R M^{\frac{1}{2}}]_i = \sum_{m=1}^{10} \left(\sqrt{\mu_i} r_{mi} \sum_{k=1}^{10} \frac{\pi_k}{\sqrt{\mu_k}} r_{mk} \right) e^{-\lambda_m t}. \quad (12)$$

Recall that the output of the system is chosen as the sum of probabilities of the five inactive states:

$$\phi(t) = \sum_{m=1}^{5} p_i(t).$$

From (12), the output $\phi(t)$ can be rewritten as

$$\phi(t) = \sum_{m=1}^{10} \alpha_m e^{-\lambda_m t}, \quad (13)$$

where

$$\alpha_m = \left(\sum_{i=1}^{5} \sqrt{\mu_i} r_{mi} \right) \left(\sum_{k=1}^{10} \frac{\pi_k}{\sqrt{\mu_k}} r_{mk} \right).$$

Recall that π and μ are the steady-state distributions for the input levels I_0 and I_1, respectively. Thus the detailed balance condition suggests that there exists a constant $c > 0$ such that

$$\begin{cases} \pi_k = c\dfrac{h(I_0)}{h(I_1)}\mu_k, \quad k = 1, \cdots, 5, \\ \pi_k = c\mu_k, \quad k = 6, \cdots, 10, \end{cases}$$

where

$$h(I) = \frac{1 + I/K_i}{1 + I/K_a}.$$

This relation and the orthogonality relation (11) suggest that for $m = 2, \cdots, 10$,

$$\begin{aligned} \alpha_m &= c \left(\sum_{i=1}^{5} \sqrt{\mu_i} r_{mi} \right) \left(\frac{h(I_0)}{h(I_1)} \sum_{k=1}^{5} \sqrt{\mu_k} r_{mk} + \sum_{k=6}^{10} \sqrt{\mu_k} r_{mk} \right) \\ &= c \left(\frac{h(I_0)}{h(I_1)} - 1 \right) \left(\sum_{i=1}^{5} \sqrt{\mu_i} r_{mi} \right)^2. \end{aligned}$$

This shows that $\alpha_2, \cdots, \alpha_{10}$ must have the same sign. This fact, together with (13), suggests that the output $\phi(t)$ must be a monotonic function. Thus an equilibrium sensory network model with detailed balance cannot perform adaptation.

References

[1] Berman A, Plemmons RJ. Nonnegative Matrices in the Mathematical Sciences. New York: Academic Press; 1979.

[2] Jia C, Chen Y. A second perspective on the Amann–Schmiedl–Seifert criterion for non-equilibrium in a three-state system. J Phys A: Math Theor. 2015;48(20):205001.

[3] Yin GG, Zhang Q. Continuous-time Markov chains and Applications: A Two-time-scale Approach. 2nd ed. New York: Springer; 2013.

[4] Jia C. Reduction of Markov chains with two-time-scale state transitions. Stochastics. 2016;88(1):73–105.
